# Preventing Neurodegenerative Memory Loss in Hopfield Neuronal Networks Using Cerebral Organoids or External Microelectronics

**DOI:** 10.1155/2017/6102494

**Published:** 2017-09-05

**Authors:** M. Morrison, P. D. Maia, J. N. Kutz

**Affiliations:** ^1^Department of Applied Mathematics, University of Washington, Seattle, WA, USA; ^2^Center for Sensorimotor Neural Engineering, University of Washington, Seattle, WA, USA

## Abstract

Developing technologies have made significant progress towards linking the brain with brain-machine interfaces (BMIs) which have the potential to aid damaged brains to perform their original motor and cognitive functions. We consider the viability of such devices for mitigating the deleterious effects of memory loss that is induced by neurodegenerative diseases and/or traumatic brain injury (TBI). Our computational study considers the widely used Hopfield network, an autoassociative memory model in which neurons converge to a stable state pattern after receiving an input resembling the given memory. In this study, we connect an auxiliary network of neurons, which models the BMI device, to the original Hopfield network and train it to converge to its own auxiliary memory patterns. Injuries to the original Hopfield memory network, induced through neurodegeneration, for instance, can then be analyzed with the goal of evaluating the ability of the BMI to aid in memory retrieval tasks. Dense connectivity between the auxiliary and Hopfield networks is shown to promote robustness of memory retrieval tasks for both optimal and nonoptimal memory sets. Our computations estimate damage levels and parameter ranges for which full or partial memory recovery is achievable, providing a starting point for novel therapeutic strategies.

## 1. Introduction

In the past few years, we have witnessed the emergence of several staggering technologies in regenerative neurobiology, including hybrid systems of neurons and semiconductor microelectronics [1, 2], brain-machine interfaces [3, 4], and the manipulation of induced pluripotent stem cells, which develop into recognizable miniature cerebral organoids [5–9]. Such advancements are likely to play a key role in future medical and rehabilitation research due to the growing numbers of traumatic brain injury incidents and the overall aging of the population, which increases the risk of dementia and neurodegenerative diseases. Even the most complex and sophisticated neuronal networks, such as the ones present in the human brain, are exposed to pathological effects that may culminate in functional losses such as motor and cognitive deficits, worse decision-making capabilities, and memory impairments. With approximately one new Alzheimer's case diagnosed every 68 seconds in the US alone, it is important to investigate in theoretical and computational settings whether minibrains, organoids, and external devices could help mitigate its most prevalent symptom: memory loss. To this end, we examine memory degradation in Hopfield neuronal networks that retrieve stored patterns from partial or noisy queues and evaluate to which extent an external auxiliary network can help restore its original functionality.

Traumatic brain injuries (TBI) and neurodegenerative diseases are among the main causes of cognitive dysfunction in humans [10–15]. Both sources of dysfunction exhibit significant presence of focal axonal swellings [16–22]. Axonal injuries hinder the information encoded in spike trains [23–25], thus leading to potentially severe functional deficits [26–32]. Challenging our understanding of the impact of axonal swellings is our inability to access small-scale injuries with noninvasive methods, the overall complexity of neuronal pathologies, and our limited knowledge of how networks process biological signals [33]. While it is difficult to diagnose and treat pathological effects on small spatial scales in vivo, recent developments in stem cell technologies might revolutionize current therapeutics [9]. Structures resembling whole organs, termed* organoids*, have been generated from stem cells for the intestine, the kidney, and, most impressively, the brain [9]. In fact, the groundbreaking work of Lancaster et al. [8] opened new routes for studying developmental diseases and degenerative conditions in minibrains [5–7]. In some cases, the tissues derived in vitro from patient cells may be used in organ replacement strategies.

Due to the complexity of the brain, however, it is not clear that the addition of a small network of external neurons or a hybrid bioelectronic system could restore the information processing capabilities required for higher cognitive functions. Moreover, there is currently a significant lack of biophysical evidence or experimental studies capable of directly addressing this issue. This motivates our development of a computational framework, using the Hopfield model for associative memory, which can provide a platform to study the conditions in which an auxiliary external network may prevent and/or reverse TBI and neurodegenerative impairments. The purpose of this study is to simulate healthy and damaged memory networks during memory retrieval tasks. In our model, we can explicitly allow an auxiliary network to communicate with the original and then delineate its effect on memory retrieval. We demonstrate that an auxiliary network can indeed mitigate the effects of memory loss due to progressive neurodegeneration of the Hopfield network.

**Figure 1:**
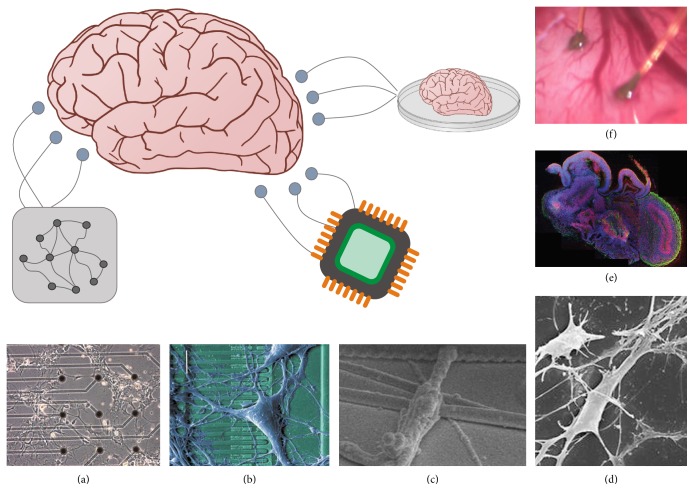
Recent technologies are enabling more sophisticated interfaces between brains and auxiliary networks which could take the form of an organoid, external electronic device, or artificial neuronal network. (a) Cultured neural cells adhere directly to a glass Petri dish with small electrodes inserted. Microelectrode array (MEA) technology is an essential component to hybrid biological-electronic systems of neuronal networks grown in vitro. Image adapted from Pizzi et al. [34]. (b) Neurons grown on a semiconductor chip, adapted from [1]. (c-d) Neuron wrapping a silicon nanowire and hippocampal neurons coupled with germanium nanowires, adapted from [35]. (e) Cerebral organoid grown in vitro from stem cells. Image adapted from Lancaster et al. [8]. (f) Microbioelectrodes implanted into a rat cortex, adapted from [36]. In all cases, the devices could advance neural prosthetics and aid damaged or aging brains to perform their original motor and cognitive functions.

Minibrains, or cerebral organoids, will likely play a critical role in our understanding of human brain development and modeling of neurodevelopmental disorders [6]. An organoid is an organized group of organ-specific cells that form an undeveloped organ in vitro [9]. Stem cells or organ progenitors are treated with growth factors and placed in conditions that allow a 3D organoid to form. Researchers treat an embryonic stem cell medium with low levels of basic fibroblast growth factor, and once a 3D aggregate of cells forms, they are transferred to a neural induction medium [5, 8]. The cerebral organoids display distinct brain regions; however, they were randomly organized and lacked the same structure as a developing human brain (Figure 1(e)) [5, 8]. In a later study, researchers formed organoids from pluripotent stem cells (PSCs) or induced PSCs (iPSCs) [9]. The organoid develops through cell sorting and spatially restricting lineage commitment in a similar manner to how the human brain develops [9]. Although cerebral organoids are currently small, they have characteristics of normal brain development and show discrete brain regions [7]. This brings hope that cerebral organoids can be useful for disease modeling, drug testing, and organ replacement. For example, Lancaster et al. used a cerebral organoid to create a more accurate model of human microcephaly [8]. Currently, cerebral organoids have some drawbacks: they can only be grown to a diameter of 4 mm due to a lack of vascularization [9], and researchers do not know to what extent electrical potential and connectivity are the same in the developing human brain and a cerebral organoid [7].

Several strategies are enabling functional interfaces between neuronal networks and electronics at various resolutions. Some of these systems are intended to interface with individual neurons. Fromherz created a hybrid system of neurons and semiconductor microelectronics by using a chip to excite and record neuronal activity (Figure 1(b)) [1]. The neurons interfaced with silicon microstructures, allowing information transfer between ion-conducting neurons and electroconducting silicon [2]. Researchers have also grown cells on graphene solution-gated field effect transistors which were able to stimulate the cells [37], and used microscale gold electrodes to communicate with rat brain neurons (Figure 1(f)) [36]. Fine-resolution stimulation has been achieved with vertical silicon nanowire probes which, with an intracellular interface, can measure a neuron's action potential [38].

Other systems are intended to interface with an entire brain region. Nanowires were successfully integrated into hippocampal regions (Figures 1(c) and 1(d)) [35]. Microelectrode array (MEA) technology can record and stimulate at the neuronal network level by sending and retrieving information at multiple network sites with a noninvasive interface [39]. This allowed stem cells that grew into neural networks to be interfaced; digital signals fed to the network via the MEAs created distinct neural output patterns (Figure 1(a)) [34]. In a later experiment, Pizzi et al. stimulated a neural network with MEAs and read the output with an artificial neuronal network [40]. Using this interface, they were able to train the neuronal network to control a robotic arm given different inputs. Current MEA directions include creating higher density MEAs, flexible electrodes, and tactics to monitor subthreshold activity [39].

In addition to interfacing with organically grown neural networks, researchers are also creating artificial neurons [41]. These artificial neurons are made with chalcogenide-based materials that change states when a current is applied; this allows them to represent a neuron's membrane potential while also being stochastic. Future studies aim to link these synthetic neurons into a network.

Brain-machine interfaces (BMIs) interpret motor intent from cortical signals and then stimulate muscles or the spinal cord. These devices have the potential to allow people with spinal cord injury to regain motor function [3]. Signals from the brain can be used to control more than just the limbs; researchers have trained monkeys to navigate a wheelchair using a BMI [42]. BMIs can also help reverse the effects of TBI by first processing neural intent from the brain and then generating targeted feedback that will help the brain execute a function, whether it is retrieving a memory or performing a motor task [4]. Connecting technology to the brain may be achieved by a recently developed microfabricated neural probe [43]. This probe is stiff for insertion into the brain but later dissolves leaving a polymeric structure, creating a suitable interface between the probe and the brain [43].

## 2. Materials and Methods

Hopfield networks of artificial neurons are the most used class of models for memory retrieval tasks [44–47]. More advanced models of memory encoding and retrieval have been developed since the pioneering work of Hopfield [48–53]. However, we focus here on a Hopfield associate memory model in order to illustrate the concepts of how an auxiliary network can be trained with the Hopfield network. Damage to axons and neuronal connections typically impairs the network's collective functionality after a critical threshold value [54]. In the worst-case scenarios, focal axonal swellings, axotomies, or cell death could block the information encoded in spike trains and effectively zero out weights in the connectivity matrix [23–25]. In this study, we couple a smaller auxiliary network to an injured Hopfield network to improve memory retrieval (Figures 3 and 4). Auxiliary neurons are connected to the original network in a sparse manner to mimic experimental constraints and trained to converge to auxiliary memory patterns. We then randomly induce injury across the original network and analyze its ability to retrieve memories with the appended external network.

### 2.1. Hopfield Model

**Figure 2:**
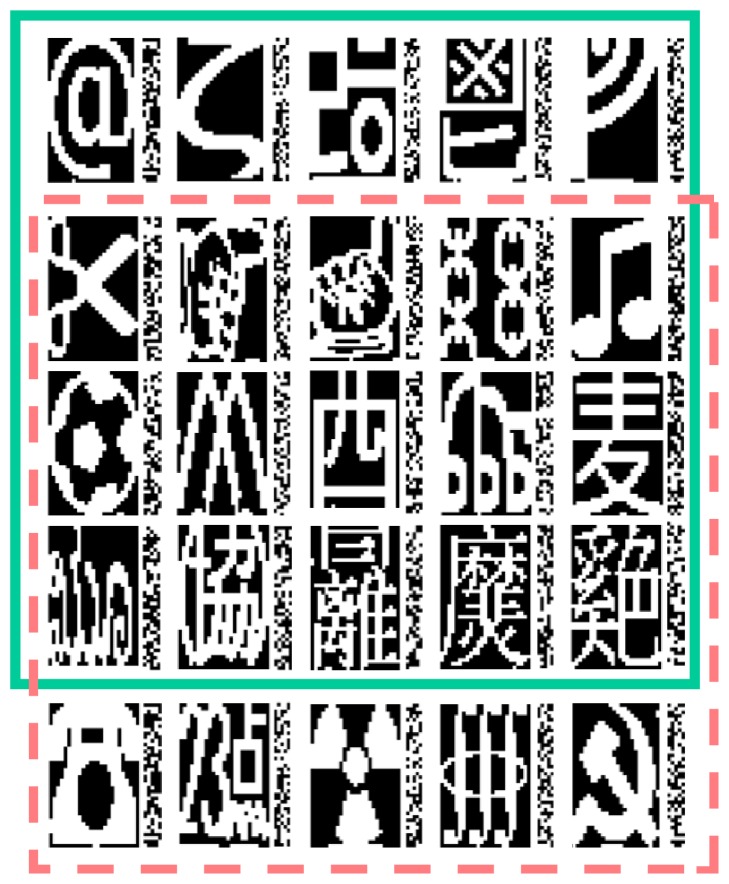
The memory sets encoded in the Hopfield networks are composed of 20 black and white pictures (40 × 25 pixels) drawn from a larger collection of Unicode symbols. The upper set (in solid green) contains an optimal set of characters that are collectively less correlated (orthogonal) to each other. The lower set (in dashed pink) shares 15 memories with the optimal set but displays a noticeably worse performance. Each memory is paired with its corresponding, calibrated auxiliary memory pattern, encoded by the external network. Auxiliary memories are significantly smaller than the original ones (40 × 5 pixels) and do not resemble the original patterns or form interpretable shapes.

In our autoassociative memory network, coupled artificial neurons respond to meaningful external queues with stable, collective activity patterns *m* ^*μ*^ (where 1 ≤ *μ* ≤ 20). The memory collection $M=\left[\begin{array}{cccc} m^{1} & m^{2} & \cdots & m^{20} \end{array}\right]$ is composed of all of the attractors of the high-dimensional dynamical system (Figure 2). In Hopfield's original formulation [44, 47], a neuron is either “on,” with state *S*_*i*_ = +1, or “off,” with state *S*_*i*_ = −1. The connectivity strength (weight) between neurons *i* and *j* is given by *w*_*ij*_ and stored in the matrix **W** = (*w*_*ij*_) = **M** **M** ^*T*^. The temporal dynamical equation for the neuronal state is given by

(1) $$\begin{array}{c} S_{i}\left(\;t+1\;\right)=g\left(\;h_{i}\left(\;t\;\right)\;\right), \end{array}$$

with input potential *h*_*i*_(*t*) = ∑_*j*≠*i*_ *w*_*ij*_ *S*_*j*_(*t*) and gain function

(2) $$\begin{array}{c} g\left(\;x\;\right)=\left\{\begin{array}{cc} +1, & x\geq 0,\\ -1, & x<0. \end{array}\right. \end{array}$$

At a given damage level, we randomly eliminate *p* (%) connections, which interferes with the network's ability to properly recover a stored memory.

### 2.2. Simulation Procedure: Hopfield Model with Auxiliary Network

To simulate a damaged brain retrieving memories, we select a memory set, encode it in a Hopfield network, induce network damage, and measure memory retrieval. Each memory is a unique binary pattern generated from a Unicode symbol (dimensions: 40 × 25 pixels), and each of the 1000 pixels represents an artificial neuron's state with value −1 (black/off) or +1 (white/on). Memory sets contain 20 symbols (see Figure 2) selected from a 30-symbol dataset. As pointed out by Gerstner et al. [47], numerical digits and alphabetical characters can be highly correlated, making it difficult for a Hopfield network to retrieve them from partial or noisy queues without some degree of confusion. To circumvent this, we optimize the 20-item memory set by choosing the most orthogonal subset (i.e., the characters that are collectively less correlated to each other). We will refer to it as the optimal network and compare it with a nonoptimal memory set that shares 15 out of 20 with it. This suffices for observing noticeable difference in performance.

### 2.3. Train Auxiliary Network

**Figure 3:**
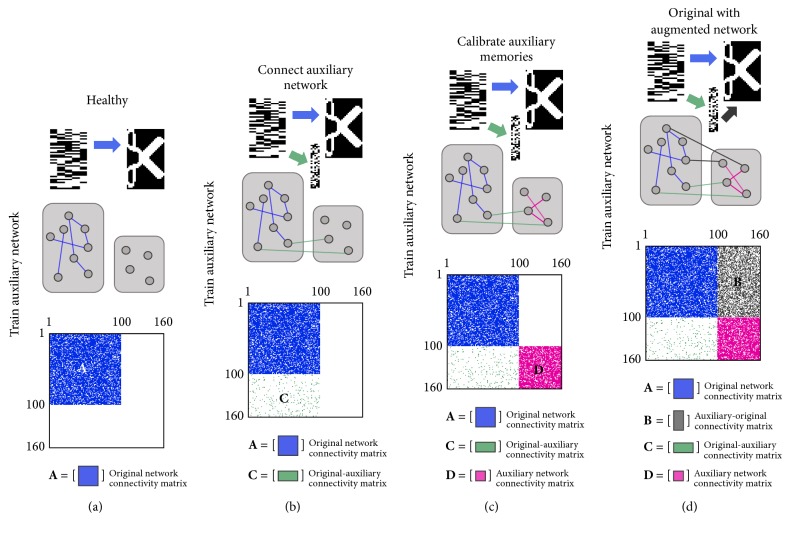
Training auxiliary network. (a) The original Hopfield network can reliably retrieve 20 stored memories. Connectivity matrix $={\tilde{W}}^{1}$. (b) An auxiliary network is sparsely connected to the original network. Connectivity matrix $={\tilde{W}}^{2}$. (c) The auxiliary network is calibrated to generate auxiliary patterns for the original memories for prevention purposes. The connectivity matrix moves from state ${\tilde{W}}^{3}$ to ${\tilde{W}}^{4}$. (d) The auxiliary network connects back to the original network. Connectivity matrix = ${\tilde{W}}^{5}$.

**Figure 4:**
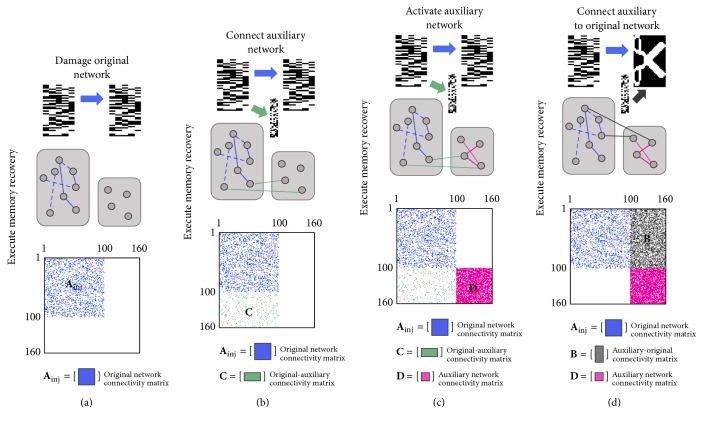
Execute memory recovery. (a) Damage in the original network severs connections and causes memory loss. Connectivity matrix $={\tilde{W}}_{inj}^{1}$. (b) Connections from the original to the auxiliary network are activated. Connectivity matrix $={\tilde{W}}_{inj}^{2}$. (c) Connections between nodes in the auxiliary network are activated and the auxiliary memory is retrieved. The connectivity matrix moves from state ${\tilde{W}}_{inj}^{3}$ to ${\tilde{W}}_{inj}^{4}$. (d) Connections from the auxiliary to the original network are activated after original-auxiliary connections are severed. Connectivity matrix $={\tilde{W}}_{inj}^{1}$. The original memory is retrieved.

In order to incorporate the auxiliary network, we extended each activity pattern to include the activity exhibited in the auxiliary network during the retrieval of a particular memory. Figures 3 and 4 and Table 1 summarize our methodology and simulation parameters. In our new model, each activity pattern is now the old memory pattern, *m*_*o*_^*μ*^, augmented with an auxiliary memory pattern, *m*_*a*_^*μ*^. Hence, ${\tilde{m}}^{\mu }=\left[\begin{array}{c} m_{o}^{\mu }\\ m_{a}^{\mu } \end{array}\right]\;\;(1\leq \mu \leq 20)$.

The memory collection is composed of these new activity patterns $\tilde{M}=\left[\begin{array}{cccc} {\tilde{m}}^{1} & {\tilde{m}}^{2} & \cdots & {\tilde{m}}^{20} \end{array}\right]$ (Figure 2). The connectivity matrix describing the connectivity weights between neurons is $\tilde{W}=\tilde{\left(w_{ij}\right)}=\tilde{M}{\tilde{M}}^{T}=\left[\begin{array}{cc} A & B\\ C & D \end{array}\right]$.

The healthy original Hopfield network encodes all 20 stored memories (Figure 3(a)); connections between neurons in this network are represented by **A**, the original network connectivity matrix. The original network is sparsely connected to a small set of auxiliary neurons represented by **C**, the original-auxiliary connectivity matrix. After calibration, the auxiliary network converges to auxiliary memory patterns (Figure 3(b)). Interconnections between neurons in this network, which now stores a set of patterns, are represented by **D**, the auxiliary network connectivity matrix. We generate the auxiliary network connectivity matrix (Figure 3(c)) with random interfacing to the original network before any damage, for prevention purposes. The auxiliary-original connectivity matrix, **B**, holds connectivity weights between auxiliary and original neurons (Figure 3(d)). The connectivity degree of the interface, that is, the fraction of novel, random connections between the two networks, may vary (5%–50%).

Just as before, the temporal dynamical equation for the neuronal state is given by

(3) $$\begin{array}{c} S_{i}\left(\;t+1\;\right)=g\left(\;h_{i}\left(\;t\;\right)\;\right), \end{array}$$

with input potential $h_{i}(t)=\sum_{j\neq i}{\tilde{w_{ij}}}^{n}S_{j}(t)$ and gain function

(4) $$\begin{array}{c} g\left(\;x\;\right)=\left\{\begin{array}{cc} +1, & x\geq 0,\\ -1, & x<0, \end{array}\right. \end{array}$$

where 1 ≤ *n* ≤ 5.

The connectivity matrix used to compute the input potential, ${\tilde{W}}^{n}$, transitions to different states to simulate the emergence and attenuation of connections within and among neurons in the original and auxiliary network. When submatrix **C** is inserted into the connectivity matrix, each original network pattern generates an auxiliary network pattern. These auxiliary patterns are recorded and then used to construct the auxiliary network connectivity matrix (submatrix **D**) via the Hopfield model.

*Network Training*. One has

(5) W~1A000⟶W~2A0C0⟶W~3A0CD⟶W~4A00D⟶W~5AB0D.

### 2.4. Execute Memory Recovery

Virtual lesions to the original network are represented by a sparsified connectivity matrix **A**_inj_ and lead to deficits in memory retrieval tasks (Figure 4(a)). But since the auxiliary network was calibrated and trained, it still retrieves its auxiliary patterns once the sparse interface **C** is activated (Figures 4(b)-4(c)), allowing for a significantly more robust system (Figure 4(d)). As a consequence, the failure rate, that is, the percent of times in which the original network fails to fully retrieve memories, decreases significantly. In what follows, we will discuss the impact of different parameters on performance: noise level (0%–50%), connectivity between the auxiliary and original networks (0%–50%), percent damage in the original network (0%–80%), and size of the auxiliary network (*N* = 200 or *N* = 400).

*Memory Recovery*. One has

(6) W~inj1Ainj000⟶W~inj2Ainj0C0⟶W~inj3Ainj0CD⟶W~inj4Ainj00D⟶W~inj5AinjB0D.

## 3. Results

### 3.1. Failure Rates and Network Performance

**Figure 5:**
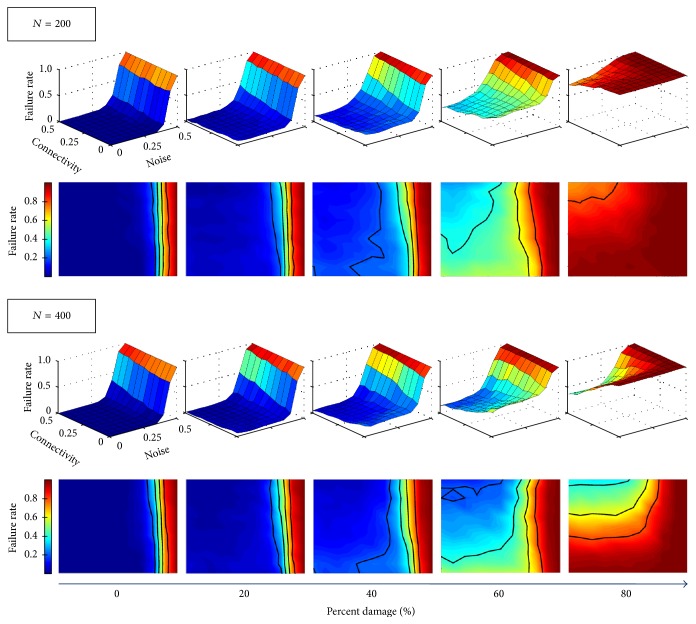
Failure rates for Hopfield networks encoded with an optimal memory set. Plots show the times (%) for which the original network fails to retrieve a memory as a function of the density of connections in **B** linking the auxiliary to the original network and the amount of initial noise in the memory cue. At higher damage levels in **A**_inj_, the auxiliary network pays off and helps decrease the failure rate. The larger auxiliary network (*N* = 400 nodes) also outperforms the smaller one (*N* = 200 nodes) at high damage levels. At low damage levels in **A**_inj_ (0%–20% damage), the auxiliary networks have little effect on original memory retrieval.

At all damage levels, failure rate increases with noise as expected (see Figure 5). For damage levels *p* ≤ 40% in **A**_inj_, the optimal network performs well and the auxiliary network has a negligible effect on failure rate. However, once *p* is beyond this value, the failure rate decreases with a denser interface in **B**, indicating that the auxiliary network, **C**, in fact prevents memory loss in the original network. As shown in Figure 5, this trend is common for both small (200 neurons) and large (400 neurons) auxiliary networks, although at *p* = 60% and 80% in **A**_inj_ the larger auxiliary network outperforms the smaller one.

**Figure 6:**
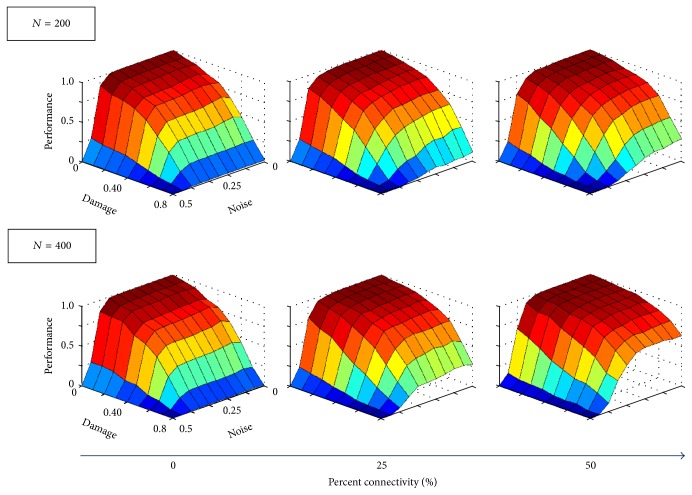
Performance for Hopfield networks encoded with an optimal memory set. Performance is now presented as a function of both damage and noise. As the connectivity level increases (increased density in **B**), performance at high damage levels also increases, indicating that denser interfaces can ameliorate the detriment caused by network damage.

Performance declines with increased damage as expected (see Figure 8); however, a denser interface in **B** improves network functionality at low noise levels *ε*. The smaller auxiliary network (200 neurons) improves performance *ε* ≤ 30% but worsens performance at larger values. The larger auxiliary network (400 neurons) also improved performance for *ε* ≤ 30% but only worsens for *ε* ≥ 40%. The shift from enhancing to undermining memory recovery may be due to the fact that, at high noise levels, the auxiliary memory might not be retrieved, therefore not feeding correct information back to the original network. Finally, Figure 6 shows how performance decreases with both damage and noise levels. Again, denser interfacing in **B** between auxiliary and original networks counteracts the detrimental effects that damage has on performance. The larger auxiliary network (*N* = 400) also enhances memory retrieval more than the smaller one (*N* = 200).

### 3.2. Comparison with Nonoptimal Memory Sets

**Figure 7:**
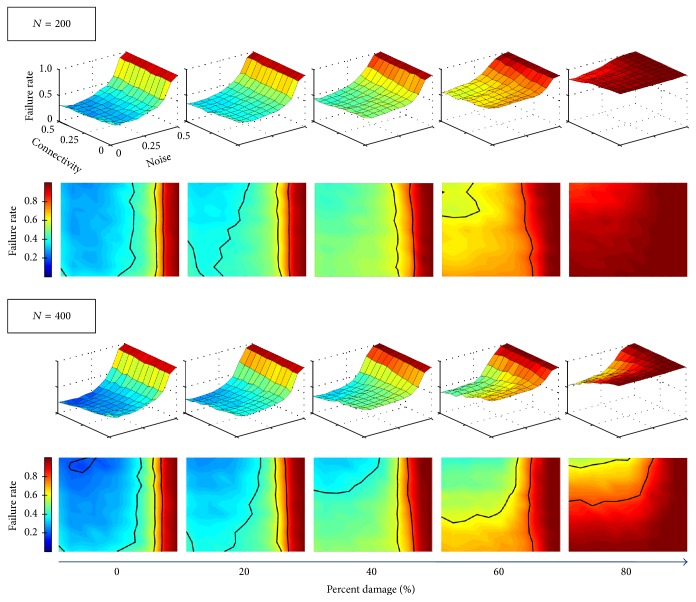
Failure rates for Hopfield networks encoded with a nonoptimal memory set. The network displays the same trends as the optimal network; the auxiliary network activity decreases failure rate particularly at higher damage levels.

**Figure 8:**
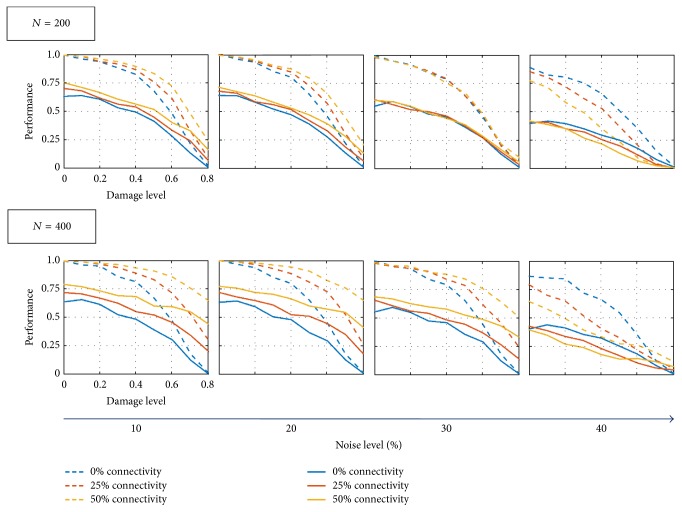
Performance for Hopfield networks encoded with optimal memory sets (dashed lines) and nonoptimal memory sets (solid lines). Original network performance increases with more auxiliary network feedback at low noise levels. For small initial noise levels (10%–30%), the original networks receiving more feedback from their auxiliary network show higher performance across all damage levels. For large noise levels (40%), the input from the auxiliary network impedes performance. The large auxiliary network increases performance at a given noise and damage level more than the small auxiliary network. The nonoptimal network performs worse than the optimal network at all damage levels and displays the same trends as the optimal network.

**Figure 9:**
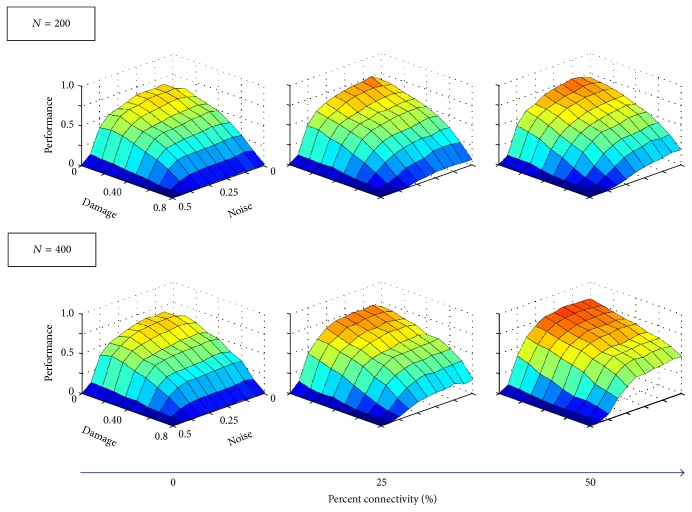
Performance for Hopfield networks encoded with a nonoptimal memory set. Performance is now presented as a function of both damage and noise. Trends are similar to the optimal network. Additionally, as connectivity increases, performance at high damage levels also increases; connectivity also ameliorates memory loss caused by damage to nonoptimal networks.

As previously mentioned, Unicode symbols may be highly correlated making it difficult for the Hopfield model to retrieve them, without confusion, from noisy or partial queues. Figures 5, 8, and 6 refer to networks that encoded an optimal memory subset. For contrasting purposes, we discuss nonoptimal memory sets which only share 15 out of 20 memories with the optimal set; the remaining elements are randomly chosen from the Unicode list of characters. As a consequence, the nonoptimal network has higher failure rates than the optimal network across all noise and connectivity levels (Figure 7). Still, the nonoptimal network shows similar trends to the optimal network; at low damage levels, the auxiliary network does not affect memory retrieval while at higher damage levels (*p* = 60% and *p* = 80% in **A**_inj_), failure rate decreases with increased connectivity. The performance trends are also analogous to the optimal network (see Figure 8), though with lower values. Again, the performance decreases with both increased damage and noise (see Figure 9) with denser interfaces and larger auxiliary networks better counteracting the detrimental effects of damage.

## 4. Discussion

In this work, we introduce a computational framework to evaluate potential enhancements in memory retrieval within a damaged Hopfield network. Axonal damage and connectivity impairments cause memory loss while communication with an auxiliary network may help prevent the loss of functionality. In our setup, the Hopfield network can tolerate significant amounts of damage before memory loss ensues, although this may vary according to the specific parameters in simulations. The beneficial/therapeutic effects of the auxiliary network are more noticeable at higher damage levels, for both optimal and nonoptimal memory sets. As a consequence, the auxiliary networks can be expected to compensate for severe deficits but not aid healthy individuals in retrieving memories.

The density of the interface between the original and external networks is a key factor for improving performance. Since there are several biological and experimental difficulties that may constrain the number of connections between the systems, it is somewhat promising that sparse connectivity suffices to calibrate complementary memories. But even if the auxiliary network needs to read activity from only a few brain regions, it must still stimulate the damaged brain in many areas.

In our setup, we interpret the original network as the human brain or one of its components and the auxiliary network taking potentially different forms such as a cerebral organoid, an artificial neuronal network, or a semiconductor chip. In order for an organoid to be useful as an auxiliary network, it must follow the same Hopfield plasticity rules that the original network follows so that connectivity will restructure during calibration. Researchers have recently grown cerebral organoids that display discrete brain regions [8, 9]. However, it is unknown whether the connectivity in these organoids can be controlled. This organic neural network must be* trainable* for it to be useful as an auxiliary network. Another obstacle to overcome is the size of the organoid. In our computational study, the larger auxiliary network aids in memory retrieval more than the small auxiliary network; the auxiliary networks were 40% and 20% the size of the original network, respectively. Current cerebral organoids have a maximum diameter of 4 mm, making them less than 2% of the size of a human hippocampus, the brain region associated with memory storage. An auxiliary network much smaller than the original network may not be capable of enhancing memory retrieval; cerebral organoids must grow larger in order to be useful auxiliary devices.

A semiconductor chip does not have the same connectivity and size constraints as a cerebral organoid. However, while a chip can reliably store and send information according to its initial programming, it is not adaptable like an organic neural network. A cerebral organoid is capable of changing over time as the human brain changes. This ability to adapt with the changing brain will perhaps allow it to consistently store and retrieve information despite memory changes in the brain.

Future work could simulate more specific or more biologically sophisticated neuronal network models and hybrid bioelectronic systems. Neurons display more complex activity than simply being “on” or “off,” although Hopfield's formulation still provides important insight into how the brain stores and retrieves memories. Additionally, novel methodologies might allow the auxiliary network to prevent memory loss with sparser auxiliary-original connectivity. This is important since experimental constraints may only allow for few connections to be made between a device and the human brain.

## 5. Conclusion

Recent technologies show promising ways to engineer biological and artificial external networks and connect them to the brain [2, 8, 9, 35, 39]. This computational study investigates whether such auxiliary apparatus could theoretically ameliorate damage in a network and improve memory retrieval. A sparse interface was sufficient to generate stable auxiliary network patterns (auxiliary memories). After the original network becomes damaged, the auxiliary network can be connected back to the original network, aiding in memory retrieval. The auxiliary network's influence is proportional to its size and connectivity to the original network, although its beneficial effects might be noticeable only at substantial injury levels.

These results imply that an auxiliary network could help a severely damaged network recover some functionality if it is calibrated before damage occurs. An auxiliary device may be able to store memories for individuals who are forecasted to suffer from Alzheimer's disease and allow them to retrieve memories despite severe neurodegeneration. An auxiliary device may also be useful to people whose lifestyle puts them at risk for TBI. Prior to getting injured, individuals could store memories in an auxiliary network and then use their device if brain damage occurs. In conclusion, auxiliary networks might be a promising road to improve brain functionality after injury or neurodegeneration.

## Figures and Tables

**Table 1:** Parameters for numerical simulations.

Number of stored memories	20 (see Figure 2)
Neurons in the original network	1000
Neurons in the auxiliary network	200 or 400
Density in **C** (connectivity to the auxiliary network)	5%
Density in **B** (connectivity to the original network)	0%–50% (5% increments)
Noise level	0%–50% (5% increments)
Sparsity in **A**_inj_ (damage level)	0%–80% (10% increments)

## References

[1] Fromherz P. (2002). Electrical interfacing of nerve cells and semiconductor chips. *A European Journal of Chemical Physics and Physical Chemistry*.

[2] Fromherz P. (2003). Semiconductor chips with ion channels, nerve cells and brain. *Physica E: Low-Dimensional Systems and Nanostructures*.

[3] Alam M., Rodrigues W., Pham B. N., Thakor N. V. (2016). Brain-machine interface facilitated neurorehabilitation via spinal stimulation after spinal cord injury: Recent progress and future perspectives. *Brain Research*.

[4] Turner D. A., Laskowitz D., Grant G. (2016). Enhanced functional outcome from traumatic brain injury with brain–machine interface neuromodulation. *Translational Research in Traumatic Brain Injury*.

[5] Bershteyn M., Kriegstein A. R. (2013). Cerebral organoids in a dish: progress and prospects. *Cell*.

[6] Brüstle O. (2013). Developmental neuroscience: Miniature human brains. *Nature*.

[7] Bae B.-I., Walsh C. A. (2013). What are mini-brains?. *Science*.

[8] Lancaster M. A., Renner M., Martin C.-A. (2013). Cerebral organoids model human brain development and microcephaly. *Nature*.

[9] Lancaster M. A., Knoblich J. A. (2014). Organogenesis in a dish: modeling development and disease using organoid technologies. *Science*.

[10] LoBue C., Denney D., Hynan L. S. (2016). Self-Reported Traumatic Brain Injury and Mild Cognitive Impairment: Increased Risk and Earlier Age of Diagnosis. *Journal of Alzheimer's Disease*.

[11] Menon D. K., Maas A. I. R. (2015). Traumatic brain injury in 2014: Progress, failures and new approaches for TBI research. *Nature Reviews Neurology*.

[12] Patterson B. W., Elbert D. L., Mawuenyega K. G. (2015). Age and amyloid effects on human central nervous system amyloid-beta kinetics. *Annals of Neurology*.

[13] Roozenbeek B., Maas A. I. R., Menon D. K. (2013). Changing patterns in the epidemiology of traumatic brain injury. *Nature Reviews Neurology*.

[14] Thies W., Bleiler L. (2013). Alzheimer’s disease facts and figures. *Alzheimer’s & Dementia*.

[15] Yue J. K., Vassar M. J., Lingsma H. F. (2013). Transforming research and clinical knowledge in traumatic brain injury pilot: Multicenter implementation of the common data elements for traumatic brain injury. *Journal of Neurotrauma*.

[16] Adalbert R., Nogradi A., Babetto E. (2009). Severely dystrophic axons at amyloid plaques remain continuous and connected to viable cell bodies. *Brain*.

[17] Daianu M., Jacobs R. E., Town T., Thompson P. M. Axonal diameter and density estimated with 7-Tesla hybrid diffusion imaging in transgenic Alzheimer rats.

[18] Krstic D., Knuesel I. (2013). Deciphering the mechanism underlying late-onset Alzheimer disease. *Nature Reviews Neurology*.

[19] Hemphill M. A., Dauth S., Yu C. J., Dabiri B. E., Parker K. K. (2015). Traumatic brain injury and the neuronal microenvironment: A potential role for neuropathological mechanotransduction. *Neuron*.

[20] Magdesian M. H., Sanchez F. S., Lopez M. (2012). Atomic force microscopy reveals important differences in axonal resistance to injury. *Biophysical Journal*.

[21] Magdesian M. H., Lopez-Ayon G. M., Mori M. (2016). Rapid mechanically controlled rewiring of neuronal circuits. *Journal of Neuroscience*.

[22] Millecamps S., Julien J.-P. (2013). Axonal transport deficits and neurodegenerative diseases. *Nature Reviews Neuroscience*.

[23] Maia P. D., Hemphill M. A., Zehnder B., Zhang C., Parker K. K., Kutz J. N. (2015). Diagnostic tools for evaluating the impact of Focal Axonal Swellings arising in neurodegenerative diseases and/or traumatic brain injury. *Journal of Neuroscience Methods*.

[24] Maia P. D., Kutz J. N. (2014). Compromised axonal functionality after neurodegeneration, concussion and/or traumatic brain injury. *Journal of Computational Neuroscience*.

[25] Maia P. D., Kutz J. N. (2014). Identifying critical regions for spike propagation in axon segments. *Journal of Computational Neuroscience*.

[26] Edlow B. L., Copen W. A., Izzy S. (2016). Longitudinal Diffusion Tensor Imaging Detects Recovery of Fractional Anisotropy Within Traumatic Axonal Injury Lesions. *Neurocritical Care*.

[27] Hånell A., Greer J. E., McGinn M. J., Povlishock J. T. (2015). Traumatic brain injury-induced axonal phenotypes react differently to treatment. *Acta Neuropathologica*.

[28] Henninger N., Bouley J., Sikoglu E. M. (2016). Attenuated traumatic axonal injury and improved functional outcome after traumatic brain injury in mice lacking Sarm1. *Brain*.

[29] Hill C. S., Coleman M. P., Menon D. K. (2016). Traumatic Axonal Injury: Mechanisms and Translational Opportunities. *Trends in Neurosciences*.

[30] Maia P. D., Kutz J. N. (2017). Reaction time impairments in decision-making networks as a diagnostic marker for traumatic brain injuries and neurological diseases. *Journal of Computational Neuroscience*.

[31] Kunert J. M., Maia P. D., Kutz J. N., Jbabdi S. (2017). Functionality and robustness of injured connectomic dynamics in *C. elegans*: linking behavioral deficits to neural circuit damage. *PLOS Computational Biology*.

[32] Rudy S., Maia P. D., Kutz J. N. (2016). Cognitive and behavioral deficits arising from neurodegeneration and traumatic brain injury: a model for the underlying role of focal axonal swellings in neuronal networks with plasticity. *Journal of Systems and Integrative Neuroscience*.

[33] Sharp D. J., Scott G., Leech R. (2014). Network dysfunction after traumatic brain injury. *Nature Reviews Neurology*.

[34] Pizzi R., Cino G., Gelain F., Rossetti D., Vescovi A. (2007). Learning in human neural networks on microelectrode arrays. *BioSystems*.

[35] Lee K.-Y., Shim S., Kim I.-S. (2010). Coupling of semiconductor nanowires with neurons and their interfacial structure. *Nanoscale Research Letters*.

[36] Andoralov V., Falk M., Suyatin D. B. (2013). Biofuel cell based on microscale nanostructured electrodes with inductive coupling to rat brain neurons. *Scientific Reports*.

[37] Hess L. H., Becker-Freyseng C., Wismer M. S. (2015). Electrical coupling between cells and graphene transistors. *Small*.

[38] Lee K.-Y., Kim I., Kim S.-E. (2014). Vertical nanowire probes for intracellular signaling of living cells. *Nanoscale Research Letters*.

[39] Kim R., Joo S., Jung H., Hong N., Nam Y. (2014). Recent trends in microelectrode array technology for in vitro neural interface platform. *Biomedical Engineering Letters*.

[40] Pizzi R. M. R., Rossetti D., Cino G., Marino D., A.L.Vescovi, Baer W. (2009). A cultured human neural network operates a robotic actuator. *BioSystems*.

[41] Tuma T., Pantazi A., Le Gallo M., Sebastian A., Eleftheriou E. (2016). Stochastic phase-change neurons. *Nature Nanotechnology*.

[42] Rajangam S., Tseng P.-H., Yin A. (2016). Wireless cortical brain-machine interface for whole-body navigation in primates. *Scientific Reports*.

[43] Sun T., Merugu S., Tsang W. M. A microfabricated neural probe with porous si-parylene hybrid structure to enable a reliable brain-machine interface.

[44] Hopfield J. J. (1982). Neural networks and physical systems with emergent collective computational abilities. *Proceedings of the National Academy of Sciences of the United States of America*.

[45] Hopfield J. J. (1984). Neurons with graded response have collective computational properties like those of two-state neurons. *Proceedings of the National Academy of Sciences of the United States of America*.

[46] Hu S. G., Liu Y., Liu Z. (2015). Associative memory realized by a reconfigurable memristive Hopfield neural network. *Nature Communications*.

[47] Gerstner W., Kistler W. M., Naud R., Paninski L. (2014). Neuronal dynamics: From single neurons to networks and models of cognition. *Neuronal Dynamics: From Single Neurons to Networks and Models of Cognition*.

[48] Garagnani M., Wennekers T., Pulvermüller F. (2008). A neuroanatomically grounded Hebbian-learning model of attention-language interactions in the human brain. *European Journal of Neuroscience*.

[49] Garagnani M., Wennekers T., Pulvermüller F. (2009). Recruitment and consolidation of cell assemblies for words by way of hebbian learning and competition in a multi-layer neural network. *Cognitive Computation*.

[50] Wennekers T., Garagnani M., Pulvermüller F. (2006). Language models based on Hebbian cell assemblies. *Journal of Physiology Paris*.

[51] Anafi R. C., Bates J. H. T. (2010). Balancing robustness against the dangers of multiple attractors in a hopfield-type model of biological attractors. *PLoS ONE*.

[52] Menezes R. A., Monteiro L. H. A. (2011). Synaptic compensation on hopfield network: implications for memory rehabilitation. *Neural Computing and Applications*.

[53] Ruppin E., Reggia J. A. (1995). Patterns of functional damage in neural network models of associative memory. *Neural Computation*.

[54] Rudy S., Maia P. D., Kutz J. N. (2016). Cognitive and behavioral deficits arising from neurodegeneration and traumatic brain injury: a model for the underlying role of focal axonal swellings in neuronal networks with plasticity. *Journal of Systems and Integrative Neuroscience*.

